# Comparison of Oral Antidiabetic Medications and Insulin Therapy for Glucocorticoid-Induced Hyperglycemia in Patients with Autoimmune Diseases

**DOI:** 10.3390/jcm14248642

**Published:** 2025-12-05

**Authors:** Shinichiro Ueno, Masataka Tajima, Kiyomi Saito, Masayuki Yoshikawa, Takeo Isozaki, Hitoshi Sato, Erika Sugiyama

**Affiliations:** 1 Department of Pharmacokinetics and Pharmacodynamics, Graduate School of Pharmacy, Showa Medical University, Tokyo 142-8555, Japan; s.ueno_88@cmed.showa-u.ac.jp (S.U.);; 2 Department of Hospital Pharmaceutics, Showa Medical University School of Pharmacy, Tokyo 142-8555, Japan; 3 Department of Pathogenesis and Translational Medicine, Graduate School of Pharmacy, Showa Medical University, Tokyo 142-8555, Japan; 4 Division of Rheumatology, Department of Medicine, Showa Medical University School of Medicine, Tokyo 142-8555, Japan

**Keywords:** glucocorticoid, glucocorticoid-induced hyperglycemia, autoimmune disease

## Abstract

**Background**: Glucocorticoid-induced hyperglycemia (GCIH) is a common adverse effect of glucocorticoid (GC) therapy. Although evidence on oral antidiabetic medications (OADMs) for GCIH is emerging, direct comparisons with insulin therapy remain limited. This study aimed to compare the efficacy and safety of OADMs and sliding scale insulin (SSI) in patients with autoimmune diseases who developed GCIH. **Methods**: We retrospectively analyzed 97 patients who developed GCIH during GC therapy equivalent to ≥20 mg/day of prednisolone. Patients were classified into SSI-only (n = 41), OADM (n = 31), and basal–bolus/basal or bolus insulin (BBI/BI) (n = 25) groups. The primary endpoint was mean preprandial blood glucose (BG), adjusted for patient characteristics. Secondary outcomes included hospital stay, hypoglycemia, insulin use, and glycated hemoglobin. **Results**: In univariate analysis, the mean preprandial BG levels during the treatment period were significantly associated with the mean initial preprandial BG levels. Adjusted mean preprandial BG during treatment did not differ significantly between the OADM and SSI-only groups, whereas the BBI/BI group had higher pre-breakfast BG (*p* = 0.016). Among first-time GC users, those in the OADM group using cyclophosphamide had significantly lower fasting BG than non-users (*p* = 0.011). **Conclusions**: In patients with autoimmune diseases receiving ≥20 mg/day GC, OADM provided glycemic control comparable to SSI with similar hypoglycemia risk. Early preprandial BG levels during the first 3 days of GC therapy may help predict glycemic outcomes. Prospective studies with standardized regimens are needed to optimize GCIH management.

## 1. Introduction

Glucocorticoid (GC) therapy is widely used in the treatment of autoimmune diseases; however, one of its notable adverse effects is glucocorticoid-induced hyperglycemia (GCIH). The mechanisms underlying GCIH include enhanced hepatic gluconeogenesis [1], reduced glucose uptake in adipose tissue and skeletal muscle [2,3], and suppressed insulin secretion through β-cell apoptosis [4]. Recent studies have demonstrated that HbA1c levels are associated with subclinical cardiovascular dysfunction and cardiovascular diseases even in patients without diagnosed diabetes [5]. Since long-term GC therapy increases the risk of cardiovascular disease due to complications such as hypertension, impaired glucose tolerance, and dyslipidemia [6], GCIH may worsen prognosis. Therefore, intervening in GCIH among patients without diabetes may improve outcomes.

In a meta-analysis by McKinnon et al., the incidence of GCIH was reported to be 32.3% among patients without diabetes receiving GC therapy [7]. Similarly, Katsuyama et al. reported that 45.7% of hospitalized patients with rheumatic or renal diseases who received GC treatment developed GCIH [8]. For GCIH management, the Joint British Diabetes Societies (JBDS) and the American Diabetes Association recommend the sulfonylurea (SU) agent gliclazide and Neutral Protamine Hagedorn (NPH) insulin [9,10]. However, both increased the insulin/GC ratio, and SU agents have been associated with hypoglycemia [11,12]. In 2012, Umpierrez et al. published practice guidelines for managing hyperglycemia in non-critically ill hospitalized patients, recommending the initiation of insulin therapy, such as basal–bolus insulin (BBI), for blood glucose (BG) levels exceeding 140 mg/dL that persist for 24–48 h during GC therapy [13]. Some studies have suggested that regimens such as BBI and NPH insulin may provide better glycemic control than sliding scale insulin (SSI) [14]. Nonetheless, due to insufficient evidence on feasible treatment options, including oral antidiabetic medications (OADMs), 62% of physicians reportedly continue to use SSI in clinical practice [15]. Recently, several reports have explored the use of non-insulin therapies, such as OADMs, for GCIH. These include studies demonstrating the effectiveness of dipeptidyl peptidase-4 (DPP-4) inhibitors in patients with renal disease [16] and reports showing improvements in postprandial hyperglycemia with nateglinide and/or acarbose in patients with rheumatoid arthritis or collagen diseases [17]. Klarskov et al. noted that, although these findings are promising, controlled trials remain limited and patient heterogeneity is considerable, emphasizing the need for studies directly comparing OADMs with insulin therapy [18].

Therefore, this study aimed to compare the efficacy and safety of SSI and OADMs, which are frequently used in clinical practice for GCIH. We retrospectively evaluated treatment courses, including BG levels, in patients with autoimmune diseases who developed GCIH and examined the influence of patient background factors.

## 2. Materials and Methods

### 2.1. Patients

From January 2018 to March 2024, we reviewed the administration records of prednisolone (PSL) and methylprednisolone (m-PSL) using the electronic medical records of patients admitted to the Departments of Rheumatology and Collagen Disease at Showa Medical University Hospital and Showa Medical University East Hospital. We retrospectively collected data on prescription periods, dosages, laboratory values, and information related to insulin and antidiabetic drug use.

Inclusion criteria were as follows:

- Received GC therapy equivalent to PSL ≥ 20 mg/day.
- Developed GCIH.
- Started any medications for GCIH.

All BG values used for GCIH determination and evaluation were measured using the Glutest Neo sensor^®^ (Sanwa Chemical Laboratory, Nagoya, Aichi, Japan), a portable glucometer for self-monitoring of capillary BG obtained from fingertip samples. HbA1c values were determined from venous blood samples by routine laboratory testing using standard automated analyzers in the hospital laboratory.

GCIH was defined as fasting BG (FBG) > 126 mg/dL or random BG > 200 mg/dL on at least two occasions after the initiation of GC therapy. The pre-breakfast BG was used as the representative FBG value to confirm GCIH.

Exclusion criteria included:

- Pregnant or breastfeeding women.
- Under 18 years of age.
- Recorded history of diabetes.
- Taking any antidiabetic medications.
- Glycated hemoglobin (HbA1c) ≥ 6.5% at the start of GC therapy.

Patients who received only SSI and no OADMs were classified as the SSI-only group. Those prescribed OADMs but no insulin other than SSI were assigned to the OADM group, while patients who received insulin preparations other than SSI were classified into the BBI/BI group.

In our department, when initiating m-PSL or PSL ≥ 0.8 mg/kg, SSI was usually started for glycemic control; otherwise, the decision to initiate OADMs or insulin regimens, along with the specifics of that treatment, was determined by the treating physicians at their discretion. No standardized criteria were applied to the choice of OADM. When BG management was initiated for patients who met the inclusion criteria, BG measurements generally began at the first pre-meal (or bedtime, etc.) after starting GC therapy of PSL ≥ 20 mg/day or m-PSL.

In the OADM and BBI/BI groups, SSI was administered as needed for hyperglycemia. SSI consisted of regular insulin (Humulin^®^ R injection), with an initial dose of 2 units for BG > 200 mg/dL, adjusted based on BG levels and individual patient conditions.

This study was approved by the Ethics Committee for Research Involving Human Subjects at Showa Medical University (approval no. 2024-093-A).

### 2.2. Study Outcomes

Extracted chart data included age, prescription medications, regular medications at hospitalization, HbA1c, and BG levels. For patients who met these criteria, additional information, such as sex, weight, height, length of hospital stay, BG measurements, SSI orders, and laboratory test results (such as estimated glomerular filtration rate [eGFR]), was obtained from electronic medical records. The primary endpoint was the mean preprandial BG (before breakfast, lunch, and dinner) after GCIH diagnosis. For the OADM group, BG data from the third day onward during monotherapy were analyzed. The initial BG was defined as the mean preprandial BG during the first 3 days of GC therapy. For patients in the BBI/BI group who also received OADMs, BG data from the period between GCIH diagnosis and OADM initiation were used. Secondary endpoints included length of hospital stay, number of patients receiving SSI, average SSI dose per administration, incidence of hypoglycemia, and change in HbA1c before and after treatment initiation.

We evaluated the changes in mean preprandial BG levels for each patient, defined as the difference between the mean during the first 3 days of GC therapy and the mean during GCIH treatment. Mean preprandial BG levels in the OADM and BBI/BI groups were compared with those in the SSI-only group. We also examined patient background factors and concomitant immunosuppressive therapies that could influence mean preprandial BG during GCIH treatment.

### 2.3. Statistical Analysis

Results are presented as medians (range, minimum–maximum). Fisher’s exact test or the Kruskal–Wallis test was used to compare patient characteristics across groups. For the primary and secondary endpoints, the SSI-only group served as the control; significant differences in the OADM and BBI/BI groups were assessed using the Steel test (continuous variables) and Fisher’s exact test (categorical variables). Changes in mean preprandial BG levels within each patient were analyzed using the Wilcoxon signed-rank test.

Univariate analyses were conducted to evaluate associations between mean preprandial BG during GCIH treatment and initial BG levels, demographic characteristics (sex, age, body mass index, HbA1c at initiation, and eGFR), medication data (PSL dose increase, initial PSL or m-PSL dose, use of cyclophosphamide [CPA], rituximab [RTX], or tacrolimus [Tac], and prior PSL use), and hospitalization duration. Among the factors mentioned above, simple linear regression analysis was performed for continuous variables, and the Wilcoxon or Kruskal–Wallis test was used for categorical variables. Next, statistically significant factors (*p* < 0.05) in the univariate analysis were selected as covariates associated with mean preprandial BG levels during the treatment period using a stepwise method. Using these covariates, analysis of covariance (ANCOVA) was conducted to compare mean preprandial BG levels during the treatment among the groups. For the adjusted means of each group, comparisons were made with the SSI-only group as the control.

The sample size was determined to achieve sufficient power (>80%) for detecting difference on BG levels of 18 mg/dL [19] at α = 0.05, assuming a standard deviation of 25 mg/dL [20]. Accordingly, the required sample size was calculated to be 31 to 32 participants per group.

A *p*-value of <0.05 was considered statistically significant. Statistical analysis was performed using JMP Student Edition 18.2.2 (SAS Institute Inc., Cary, NC, USA).

## 3. Results

### 3.1. Patient Characteristics and GCIH Treatment

**Figure 1:**
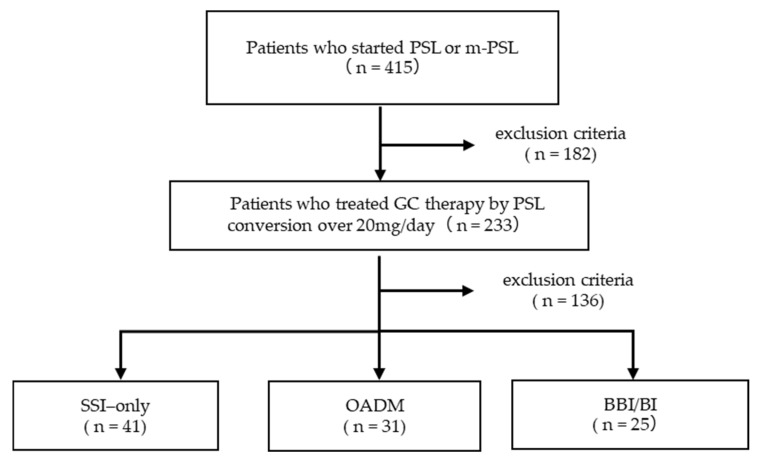
Study flow chart.

During the study period, 415 patients received PSL or m-PSL. Pregnant and breastfeeding patients were not included. A total of 182 patients were excluded: 1 minor, 21 patients who were receiving diabetes medications at admission, 70 with HbA1c ≥ 6.5% at the start of GC therapy, 23 whose HbA1c was not measured at baseline, 1 with a recorded history of diabetes, and 66 receiving <20 mg/day PSL equivalent (Figure 1). Of the 233 patients who met the inclusion criteria, 136 were excluded: 132 who did not receive either oral antidiabetic drugs or insulin, and 1 who was not diagnosed with GCIH. Consequently, 100 patients (42.9%) required treatment for GCIH (median day 1, range −29 to +7). In addition, three patients had insufficient pre-breakfast FBG data. Thus, a total of 97 patients were included in the final analysis. Among these, 41 patients were treated only with SSI (SSI-only group), 31 received OADMs (OADM group), and 25 were prescribed insulin regimens other than SSI (BBI/BI group).

The clinical characteristics at admission and post-admission pharmacotherapy for each group are summarized in Table 1. There were no significant differences among the groups in patient background, such as age, sex, renal function, HbA1c, continuation of PSL therapy, or comorbidities. All patients received PSL as part of GC therapy, and the initial daily dose did not differ significantly among the three groups. In addition, GC pulse therapy with m-PSL (1000 or 500 mg/day for 3 days) was administered to >80% of patients in all groups after hospitalization. During hospitalization, an increase in the daily dose of PSL associated with the treatment of the primary disease differed significantly among the groups (*p* = 0.035) and was more frequent in the BBI/BI group. Among patients in the OADM group, 25 received DPP-4 inhibitors, 5 received α-glucosidase inhibitors, and 1 received a sodium–glucose cotransporter 2 inhibitor, all at standard doses. In the BBI/BI group, an average of 1.56 insulin preparations (range, 1–3) per patient was used during the observation period. Specifically, 17, 14, 2, 4, and 2 patients received insulin lispro, regular insulin (Humulin^®^ R), insulin glargine, extended-release insulin glargine (Lantus^®^ XR), and insulin degludec (Tresiba^®^), respectively. Furthermore, immunosuppressive therapies other than GC were also used in all groups for disease management, with no significant differences in the types of medications administered.

### 3.2. Initial and On-Treatment BG During GCIH Therapy

**Figure 2:**
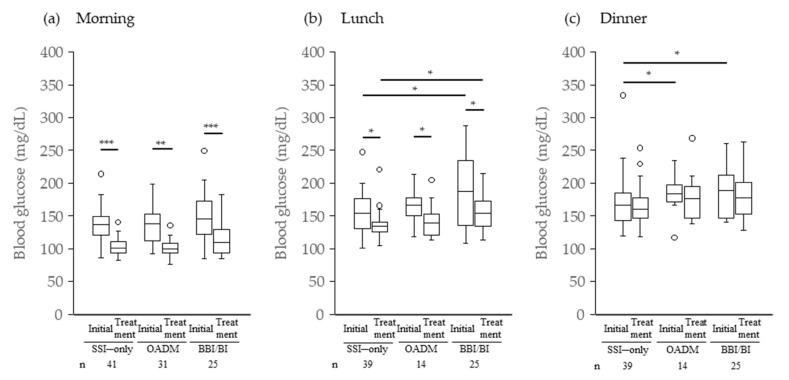
Mean preprandial blood glucose levels after starting GC therapy. * *p* < 0.05 Steel test or Wilcoxon signed-rank test; ** *p* < 0.005 and *** *p* < 0.0005 Wilcoxon signed-rank test; Initial, mean preprandial blood glucose levels for the first 3 days; Treatment, mean preprandial blood glucose levels on-treatment GCIH; GC, glucocorticoid; GCIH, glucocorticoid-induced hyperglycemia.

Figure 2 shows the mean preprandial BG levels for each group during the first 3 days after the start of GC therapy (initial BG) and throughout the GCIH treatment period (on-treatment BG). There were no significant differences in the mean initial pre-breakfast BG among the three groups, and approximately 80% of patients in each group had FBG values above the standard, that is, 110 mg/dL (78.0%, 77.4%, and 84.0% for SSI only, OADM, and BBI/BI, respectively) [21]. In contrast, compared with the SSI-only group, the OADM group showed significantly higher mean initial pre-dinner BG levels (*p* = 0.043) (Figure 2b). Furthermore, in the BBI/BI group, significantly higher mean preprandial BG levels were observed before lunch as well as before dinner compared with the SSI-only group (before lunch: *p* = 0.040; before dinner: *p* = 0.038) (Figure 2b,c).

The number of days from the start of GC until it was judged as GCIH was 2 (range, 1–43) in the SSI-only group and 2 (1–17) in the OADM and BBI/BI groups (median, min-max). Patients judged to have GCIH within 3 days after starting GC therapy were 70.7% (29/41), 80.6% (25/31), and 84.0% (21/25) for SSI only, OADM, and BBI/BI, respectively. During the treatment period for GCIH, the mean preprandial BG levels significantly decreased before breakfast and lunch compared with the initial values, although individual differences were observed (Figure 2a,b). However, no significant decrease was observed in any group before dinner (Figure 2c). In addition, the mean pre-lunch BG level in the BBI/BI group was significantly higher than that in the SSI-only group (*p* = 0.011) (Figure 2b).

### 3.3. Factors Associated with On-Treatment Preprandial BG

Univariate analysis showed that the mean preprandial BG levels during the treatment period were significantly and positively correlated with the mean initial preprandial BG levels (before breakfast: *r* = 0.326; *p* = 0.001; before lunch: *r* = 0.425; *p* = 0.0001; before dinner: *r* = 0.632; *p* < 0.0001). In addition, an increase in PSL dosage during hospitalization was identified as a significant factor associated with higher mean on-treatment BG levels before breakfast (*p* = 0.012). Age (*r* = 0.268; *p* = 0.017) and HbA1c at admission (*r* = 0.231; *p* = 0.041) were also significant factors associated with higher on-treatment pre-dinner BG levels. Next, we examined the effects of these factors on mean preprandial BG levels during the on-treatment period using a stepwise method. As a result, HbA1c at admission was excluded as a contributing factor for on-treatment pre-dinner BG (*p* = 0.306). The mean preprandial BG levels in the OADM group, adjusted for the aforementioned factors as covariates, showed no significant differences compared with the SSI-only group at any time point. In contrast, in the BBI/BI group, pre-breakfast BG levels were significantly higher than those in the SSI-only group (*p* = 0.016) (Table 2).

### 3.4. CPA Use and FBG in First-Time GC Recipients

**Figure 3:**
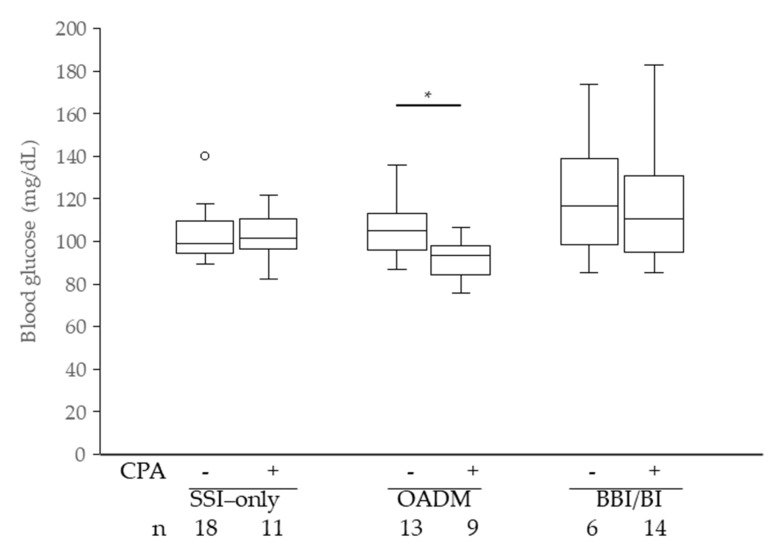
On-treatment mean pre-breakfast blood glucose levels, excluding patients who used PSL before hospitalization. PSL, prednisolone; CPA, cyclophosphamide; * *p* < 0.05 Wilcoxon rank-sum test.

Figure 3 shows the effects of CPA treatment on FBG levels in patients newly initiated on GC therapy. In the OADM group, patients treated with CPA had significantly lower mean FBG levels than those not treated with the drug (*p* = 0.011). No significant differences were observed in the SSI-only or BBI/BI groups.

### 3.5. Secondary Outcomes: Hospital Stay and Hypoglycemia

The length of hospital stay was similar between the OADM group and the SSI-only group, but was significantly longer in the BBI/BI group compared with the SSI-only group (*p* = 0.048). No significant differences in the incidence of hypoglycemia (<70 mg/dL) during the GCIH treatment period were observed between the SSI-only group and either the OADM or BBI/BI group (Table 3). In the OADM group, all four patients who experienced hypoglycemia were receiving CPA, and a significant association was observed between CPA use and hypoglycemia incidence (*p* = 0.016, Fisher’s exact test). In contrast, among patients not receiving CPA, the incidence of hypoglycemia tended to be lower in the OADM group than in the SSI-only group (*p* = 0.063, Fisher’s exact test).

### 3.6. Secondary Outcomes: Comprehensive Glycemic Control

During the observation period, the number of patients who required insulin administration based on the insulin scale was significantly lower in the OADM group (*p* < 0.0001). However, the mean insulin dose in both the OADM and BBI/BI groups did not differ significantly from that in the SSI-only group. We examined 58 patients whose HbA1c was measured during outpatient visits after discharge (median day 15, range 2–48). No significant differences were observed in the change in HbA1c values from admission to post-discharge among the treatment groups, although considerable individual variation was noted. In univariate analysis, the initial pre-dinner BG level (*p* = 0.022) and the mean pre-dinner BG level during the treatment period (*p* = 0.0533) were associated with changes in HbA1c. Given that these two factors were correlated (*r* = 0.616; *p* < 0.0001), ANCOVA was conducted using the initial pre-dinner BG level as a covariate. After adjustment, the OADM group showed a modest but statistically significant reduction in HbA1c compared with the SSI-only group (*p* = 0.034). Detailed results are provided in Supplementary Table S1.

## 4. Discussion

In this study, among patients with autoimmune diseases who were administered PSL at doses of ≥20 mg/day, approximately 40% required treatment for GCIH. This incidence was comparable to that reported in hospitalized patients with rheumatic or kidney diseases who received GC treatment (45.7%) [8]. The median time to GCIH determination was 2 days, and many cases (75/97) were identified within the first 3 days of GC initiation. Kuroki et al. reported that pulse therapy or GC therapy with ≥40 mg/day of PSL significantly increased FBG levels within 3 days in patients with collagen disease [22]. Similarly, in a study of patients with inflammatory rheumatic diseases, 42% developed GCIH within 5 days of GC initiation, with many showing elevated BG on day 3 [23]. Therefore, the findings of this study are consistent with previous reports.

In the initial stages of GC therapy, preprandial BG levels were higher in the OADM and BBI/BI groups than in the SSI-only group, which may have influenced subsequent treatment strategies for GCIH. According to the JBDS guidelines, BG should be measured before lunch or dinner, and if hyperglycemia persists, glimepiride, an SU, is recommended [9]. In addition, Perez et al. proposed the use of glinides, SUs, or DPP-4 inhibitors for patients with preprandial BG ≤ 200 mg/dL, regardless of GC type [24]. Thus, although the recommended criteria for OADM treatment are not clearly defined, the treatment decisions in this study appear to align with previous recommendations. Moreover, reduced renal function (eGFR ≤ 40 mL/min), a known risk factor for GCIH, was more prevalent in the BBI/BI group (12/25) [8]. Given that some OADMs require dose adjustment or are contraindicated in renal impairment, insulin-based therapy may have been selected in these patients [25]. This heterogeneity of the type of treatments to lower BG levels in this study is one of the limitations, while allowing comparisons across treatment groups, i.e., SSI-only, OADM, and BBI/BI groups.

In this study, DPP-4 inhibitors were the most commonly prescribed OADM for GCIH (80.6%, 25/31). Previous reports indicated that metformin and glimepiride were used in approximately 30% of patients with GCIH between 2013 and 2023 [26]. One possible explanation for the predominant use of DPP-4 inhibitors is their favorable safety and pharmacologic profile. SU agents have long durations of action and pose a risk of hypoglycemia, while metformin has a delayed onset of action in patients with GCIH [11,24]. Experimental data also support the use of DPP-4 inhibitors; Uto et al. (2021) reported that dexemethasone-treated mice showed decreased glucagon-like peptide-1 and increased DPP-4 levels in glucose tolerance tests, suggesting the potential utility of DPP-4 inhibitors for GCIH [27]. For this reason, DPP-4 inhibitors have gained attention as a treatment option for GCIH [11,28,29], and were mainly chosen at physicians’ discretion in our study. Previous reports have discussed a possible association between DPP-4 inhibitors and the risk of certain autoimmune and inflammatory bowel diseases [30]. However, since this study focused on patients who had already developed autoimmune diseases, the impact of DPP-4 inhibitors remains unclear. Notably, in this study, the number of patients in the OADM group requiring an increased PSL dose did not differ significantly from that in the SSI-only group (*p* = 0.241), and DPP-4 inhibitors were not associated with worsening of the underlying disease.

This study examined the mean preprandial BG levels during GCIH treatment. Multivariate analysis revealed that initial preprandial BG levels after GC initiation, PSL dose escalation during hospitalization, and age were significant factors. Notably, the initial preprandial BG levels showed the strongest association, indicating that higher BG levels at the start of GC therapy were associated with higher mean on-treatment BG levels. Therefore, monitoring BG levels during the first 3 days of GC therapy may help identify patients who require intensive intervention. Other factors influencing the mean preprandial BG level during treatment were age and PSL dose increase. Age is a known risk factor for GCIH [8], likely due to impaired glucose tolerance and decreased pancreatic β-cell function associated with aging. Therefore, it is reasonable to consider age as a factor influencing the mean preprandial BG level during treatment. Furthermore, an increase in PSL dose during treatment generally reflects insufficient control of the underlying disease activity. In patients with rheumatoid arthritis, inflammatory cytokines (tumor necrosis factor-α and interleukin-6) and C-reactive protein have been shown to be positively correlated with the insulin resistance index (homeostasis model assessment) [31]. In patients with rheumatoid arthritis and systemic lupus erythematosus, the factors associated with insulin resistance differ, and BG fluctuations have been reported to vary depending on the disease activity and degree of inflammation [32]. The present study included patients with a wide spectrum of autoimmune diseases, which makes it difficult to generally discuss the mechanisms of action of OADMs or insulin regimens on GCIH.

When the obtained effect factors were adjusted for covariates and the mean on-treatment preprandial BG levels were examined, the pre-dinner BG levels decreased to similar levels in the OADM and BBI/BI groups compared with the SSI-only group. However, the BBI/BI group maintained significantly higher pre-breakfast BG levels during treatment than the SSI-only group. Merkofer et al. reported that patients receiving GC therapy who required BBI had higher FBG levels than those managed with regular bolus insulin [33]. These findings suggest that GCIH may have two types: one that requires BBI and another that can be managed with low-dose insulin or without insulin. In this study, six patients received BBI, indicating that the BBI/BI group included patients with more severe GCIH than the other groups.

It is known that even a relatively low dose of PSL (7.5 mg) administered for 2 weeks can induce insulin resistance [34]. Therefore, in this study, we found that concomitant use of CPA with GC significantly decreased mean FBG levels in the OADM group, specifically among patients without prior PSL exposure who were newly initiated on PSL therapy (Figure 3). CPA is known to suppress the production of insulin antibodies, thereby relieving the inhibition of insulin activity by these antibodies and lowering BG levels [35,36]. Among the patients in the OADM group receiving CPA, nine were taking DPP-4 inhibitors. A previous study reported that patients receiving DPP-4 inhibitors for GCIH had lower morning FBG levels, likely due to their effect on nocturnal insulin secretion [37]. Taken together, these findings suggest that CPA may enhance the action of insulin secreted at night, particularly in patients receiving DPP-4 inhibitors, resulting in lower pre-breakfast FBG levels. Alternatively, gastrointestinal side effects caused by CPA may have affected food intake; however, since the comparison was based on average BG levels during the treatment period, this contribution is likely minimal.

In this study, the use of OADMs did not appear to prolong hospitalization or increase the incidence of hypoglycemia (Table 3), suggesting that OADM-based management did not adversely affect the treatment course of the underlying disease. In contrast, the significantly longer hospital stay observed in the BBI/BI group may reflect the greater severity of the underlying disease in this group. Notably, all patients who experienced hypoglycemia in the OADM group were also receiving CPA. Although the clinical significance of the interaction between OADMs and CPA remains unclear, caution may be warranted when combining CPA with OADMs, particularly DPP-4 inhibitors, because of the potential risk of morning hypoglycemia. Furthermore, the requirement for SSIs can be regarded as an indicator of hyperglycemia during treatment. In this study, significantly fewer patients in the OADM group required SSIs, suggesting that OADM-based management provided satisfactory glycemic control for many patients.

HbA1c reflects long-term average BG levels over approximately 3 months and recent BG level fluctuations [38,39]. Therefore, the change in HbA1c from admission to the first outpatient visit after discharge is considered to reflect the impact of GCIH on BG levels during hospitalization. In this study, elevated initial or on-treatment pre-dinner BG levels were associated with greater increases in HbA1c. Moreover, when comparing treatment groups, the OADM group showed a significant decrease in HbA1c after discharge compared to the SSI-only group, suggesting that the OADM group was more effective in controlling high BG levels during GC treatment, although the absolute difference in HbA1c was modest.

This study has some limitations worth noting. First, it was a single-center, retrospective study with a relatively small sample size and a wide spectrum of autoimmune diseases with different pathogenesis. In addition, there was significant heterogeneity of glucose-lowering treatments among patients, including the type of OADMs and insulin regimens, which may have influenced glycemic outcomes. Second, the timing of BG measurements varied among patients. Third, we were unable to collect sufficient data on postprandial BG levels, which are known to increase with GCIH. Finally, the number of patients who had their HbA1c measured after discharge was limited, raising the possibility of selection bias in assessing the impact of GCIH on HbA1c. The effects of Tac and mycophenolate mofetil, which are other drugs that affect BG levels and are used in conjunction with CPA, could not be evaluated because of the limited number of patients receiving them. Similarly, subgroup analyses of OADM agents and insulin formulations, including comparisons of their types and doses, were not feasible due to the limited sample size.

## 5. Conclusions

In patients with autoimmune diseases receiving GC therapy at a dosage of ≥20 mg/day, OADM treatment appeared comparable to SSI treatment in terms of glycemic control and adverse events such as hypoglycemia. However, caution is warranted regarding the risk of hypoglycemia when OADMs are combined with CPA. Furthermore, the mean preprandial BG levels during the first 3 days of GC therapy were associated with the severity of GCIH and the choice of treatment strategy, suggesting their potential utility in predicting subsequent glycemic control after GCIH onset. Future prospective clinical trials with standardized treatment regimens and BG monitoring schedules will be necessary to establish a more robust evidence base for GCIH treatment.

## Figures and Tables

**Table 1:** Characteristics of patients.

	SSI-Only	OADM	BBI/BI	*p*-Value
Patients (n)	41	31	25	
Sex (male), n	9	11	12	0.083
Age (years old), median (range)	68 (19–89)	69 (19–92)	78 (37–90)	0.154
BMI (kg/m^2^), median (range)	20.3 (13.4–28.0)	21.1 (15.3–32.0)	21.9 (16.2–30.2)	0.112
HbA1c (%), median (range)	5.9 (3.8–6.4)	5.9 (2.9–6.4)	6.0 (4.4–6.4)	0.301
eGFR (mL/min/1.73 m^2^), median (range)	82.1 (6.5–171.9)	68.5 (21.4–157.9)	46 (9.3–175.9)	0.052
PSL user (n)	12	9	5	0.706
Disease (n)				0.146
MPA/GPA/EGPA	4/2/2	2/3/0	8/1/1	
PM/DM	11	8	6	
AOSD	1	3	2	
SLE	4	8	0	
RA	3	1	3	
Others	14	6	4	
PSL start daily dose (mg/day), median (range)	45 (20–80)	45 (20–58)	45 (20–90)	0.731
Increased PSL	11	4	11	0.035 *
m-PSL 1000 mg/500 mg	19/14	6/19	9/12	0.146
Immunosuppressive therapy	26	24	22	
CPA	15	12	14	0.288
Tac	15	14	8	0.578
IVIG	7	5	7	0.445
RTX	5	6	4	0.725
MTX	2	3	4	0.334
MMF	4	1	3	0.415
CyA	2	1	3	0.486

BMI, Body mass index; HbA1c, Glycated hemoglobin; eGFR, Estimated glomerular filtration rate; PSL, prednisolone; MPA, Microscopic polyangiitis; GPA, Granulomatosis with polyangiitis; EGPA, Eosinophilic granulomatosis with polyangiitis; PM/DM, Polymyositis/dermatomyositis; AOSD, Adult-onset Still’s disease; SLE, Systemic lupus erythematosus; RA, Rheumatoid arthritis; Tac, Tacrolimus; CPA, Cyclophosphamide; IVIG, Intravenous immunoglobulin; RTX, Rituximab; MTX, Methotrexate; MMF, Mycophenolate mofetil; CyA, Cyclosporine A; * *p*-value < 0.05 Fisher’s exact test.

**Table 2:** Adjusted mean (±SE) of on-treatment mean preprandial blood glucose levels by treatment group, analyzed via ANCOVA with each significant factor as a covariate.

		LS Mean ± SE (mg/dL)	95% CI	*p*-Value
Morning	SSI-only	104.17 ± 2.57	99.07–109.28	
	OADM	99.72 ± 3.83	91.99–107.46	0.550
	BBI/BI	114.89 ± 2.99	108.97–120.82	0.016 *
Lunch	SSI–only	139.09 ± 3.52	132.08–146.10	
	OADM	142.49 ± 5.75	131.04–153.93	0.843
	BBI/BI	149.91 ± 4.47	141.00–158.82	0.127
Dinner	SSI-only	169.49 ± 3.85	161.81–177.16	
	OADM	175.76 ± 6.34	163.12–188.40	0.628
	BBI/BI	173.17 ± 4.82	163.56–182.77	0.795

ANCOVA, analysis of covariance; SE, standard error; LS, least squares; CI, confidence interval; SSI, sliding scale insulin; OADM, oral antidiabetic medication; BBI, basal–bolus insulin; BI, basal or bolus insulin; * *p*-value < 0.05 Dunnett’s test.

**Table 3:** Secondary endpoints.

	SSI-Only	OADM	*p*-Value	BBI/BI	*p*-Value
Hospital stay (day)	48 (9–194)	39 (7–78)	0.294	65 (26–208)	0.048 *
Change in HbA1c (%)	0 (−1.6–1.5) (n = 22)	−0.05 (−1.1–1.8) (n = 24)	0.896	−0.3 (−1.1–0.7) (n = 12)	0.540
Hypoglycemia (n)	7	4	0.747	6	0.535
without CPA	5	0	0.063	3	0.672
Patients using SSI (n)	38	8	<0.0001 ***	20	0.242
Average SSI dose (unit/time)	2 (1.6–6)	2.1 (2–4.7)	0.694	2.5 (2–4.7)	0.094

* *p*-value < 0.05 Steel test; *** *p*-value < 0.0005 Fisher’s exact test; SSI, sliding scale insulin; OADM, oral antidiabetic medication; BBI, basal–bolus insulin; BI, basal or bolus insulin; CPA, cyclophosphamide; HbA1c, glycated hemoglobin.

## Data Availability

The data are not publicly available due to privacy restrictions.

## Supplementary Materials

The following supporting information can be downloaded at https://www.mdpi.com/article/10.3390/jcm14248642/s1, Table S1: Adjusted mean (±SE) change in HbA1c by treatment group, analyzed using ANCOVA with mean initial pre-dinner blood glucose levels as a covariate.

## Abbreviations

The following abbreviations are used in this manuscript:

BG	Blood Glucose
BBI	Basal–Bolus Insulin
BI	Basal or Bolus Insulin
CPA	Cyclophosphamide
eGFR	Estimated Glomerular Filtration Rate
FBG	Fasting Blood Glucose
GC	Glucocorticoid
GCIH	Glucocorticoid-Induced Hyperglycemia
HbA1c	Glycated Hemoglobin
JBDS	Joint British Diabetes Societies
OADM	Oral Antidiabetic Medication
PSL	Prednisolone
SU	Sulfonylurea
SSI	Sliding Scale Insulin
