# Supplementary material for: Comparison of Oral Antidiabetic Medications and Insulin Therapy for Glucocorticoid-Induced Hyperglycemia in Patients with Autoimmune Diseases
Source: J Clin Med. 2025 Dec 5;14(24):8642. doi: 10.3390/jcm14248642 (PMC12733825; doi:10.3390/jcm14248642)
Supplement: Supplementary file 1 [file jcm-14-08642-s001.zip › jcm-4004491-supplementary.pdf]

Supplementary Table S1. Adjusted mean (±SE) change in HbA1c by treatment group, analyzed using ANCOVA with mean initial pre-dinner blood glucose levels as a covariate

	LS Mean±SE	95% CI	*p*-value
SSI–only	0.120±0.124	-0.13–0.37	
OADM	-0.417±0.175	-0.77– -0.06	0.036*
BBI/BI	-0.287±0.164	-0.62–0.04	0.107

ANCOVA, analysis of covariance; SSI, sliding scale insulin; OADM, oral antidiabetic medication; BBI, basal–bolus insulin; BI, basal or bolus insulin; HbA1c, glycated hemoglobin; * p<0.05 Dunnett's test.
